# Monthly Periscope

**Published:** 1854-02

**Authors:** 


*Monthly Periscope.*—When the editor of the Medico-Chirurgical Review said the world would have been the better, or that it would have witnessed less disease had there never been a doctor, he said a severe and dangerous thing. It was severe because it was in some measure truthful; it was dangerous because it contained just truth enough to make it dangerous. Half the truth is not unfrequently a greater error than an entire falsehood—or if not a greater error, it does more harm.

A falsehood can be contradicted and disproved—a sophistry requires argument and an array of facts to controvert it. We are obliged to admit that "there is some truth in it," and the unthinking mind does not recognize the fact that this modicum of truth is the vehicle of the falsehood.

In a conversation—the other evening—with one who holds the interests of the profession very near to him, who has lived long enough, and thought and studied sufficiently, to place him on a high scale of medical attainment, he lamented what he considered the progressive deterioration of the profession, as manifested in the smaller number of men of talent who enter it now, as compared with his earlier days. It is quite common to hear such remarks from the older members of the profession, sometimes, as in the case of our friend, expressed quietly, and in tones of regretful conviction, and sometimes loudly and angrily proclaimed from the lecturer's desk, coupled with declamation and fierce invective on the degeneracy of the craft. We have often expressed our dislike to the denunciations of these latter prophets of evil. To those who sincerely believe that medicine is on the retrograde we would commend the following page of statistics which we take from the Boston Medical and Surgical Journal:

*Mortality of the Town of Hampton, N. J.*—Rev. Mr. Hartwell, of Hampton, has furnished for the Portsmouth Morning Chronicle, some interesting statistics respecting the mortality of that town. It appears that during the last year, the number of deaths was 34—being 1 in 35 of the inhabitants. This is a very great mortality for the town, and more than has died in any year for ninety-nine years. In 1734, 68 died, 61 by throat distemper. In 1754, 51 deaths occurred, 37 by throat distemper. No epidemic has prevailed during the last year, as the following list will show: Of old age, 6; consumption 7; lung fever, 6; typhoid fever, 3; convulsion fits, 2; disease of kidneys, 1; disease of heart, 1; erysipelas, 1; abdominal abscess, 1; small pox, 1; drowned, 1; scalded, 1; nervous disease, 1; cholera infantum, 1.

It will be seen by the above, that lung diseases have prevailed largely over all others, proving that a damp atmosphere is unfavorable to those who may be predisposed to lung difficulties. The ages were as follows:—Between 90 and 100, 1; 80 and 90, 6; 70 and 80, 3; 60 and 70, 1; 50 and 60, 1; 40 and 50, 7; 30 and 40, 1; 20 and 30, 4; 10 and 20, 2; 5 and 10, 0; 1 and 5, 5; under 1, 2. If we should return the ages exactly, the united ages of ten of those who have died this year, would be 823 years and 6 months, averaging 82 years and 4 months to each one.

In 120 years, last past, commencing in 1734, 482 have died in town over 70 years old. The whole number which have died in 120 years, is 1847, not including any who have been lost at sea or in the wars. In these 1847 deaths, 230 have died between 70 and 80 years old—males, 90; females, 110. Between 90 and 100, 49 have died—males, 19; females, 30. Over 100, 1, and this a female, who died aged 104 years and 17 days.

In addition to the statistics which we have given above, from Mr. Hartwell's praiseworthy researches, he has gathered from them the following gratifying results, which corroborate the truth of Prof. Clark's affirmative answer to the question—"Has medical science lengthened human life?" Mr. H. says—"Another fact appears in examining the records of the town, that the average of life has been much lengthened in the period of 120 years. Diseases were once less complicated and severe than now, much less of sickness in proportion to the whole number of inhabitants, and anything like a contagion or epidemic rarely known. When such did occur, as in the throat distemper of 1734 and 1754, a large proportion of all attacked with it died; and those who died of the epidemic, constituted nearly all who died in 1734, there being 60 deaths by this disease, and 8 deaths by all other diseases. In 1754, in 51 deaths, 37 died of throat distemper, and 14 by all other diseases; showing that in the latter period this disease was better managed on the part of medical attendants than in the first instance. Taking the record of the town, and the testimony of several of the most aged persons, it is very evident that there is much more sickness in proportion, and that, too, with complicated and dangerous maladies, and still many less in proportion die!—showing conclusively that life is being lengthened, and that medical science is on the advance, and on the part of medical attendants there is much more ability to 'grapple with fell diseases and conquer.' And whatever credit, in view of these facts, comes to the doctors, comes to the regular or allopathic course of treatment, for quackery of any sort in medicine has but little prevailed here, and in most instances where quacks and uneducated men have been employed, the diseases they have been called to treat have proved fatal in their hands."

One such collection of evidence is worth a book of lamentations. It was very justly remarked by another who was present at the conversation which has suggested these remarks, that with increasing years and knowledge, the men and facts of the present grow small. The teachers who signed our diplomas were, to our youthful eyes, men of wonderous attainment and capacious intellect. This is because they were really far our superiors at that time' and the impression of their greatness is indelible. But when the former tyro is himself a savant when he sees men whom he knows to be his inferiors occupying positions once held by those who seemed so great to him in youth, he looks upon it not as it really is, a change for the better in himself, but as a change for the worse in those who fill the places which he once reverenced. On the other hand, those still burning with the fire of youth, mistake not unfrequently, their individual advancement in knowledge for a real progress in the profession. But this can do no harm save as it feeds the vanity of youth. It stimulates research, and while it may deceive with a false hope it never utterly falsifies that hope. The sower of the seed returns at last bearing with him his sheaves. It is urged that the profession has fallen off in the public estimation—that quackery is more rampant than ever—that intellectual advancement does not increase the confidence felt in legitimate medicine.

We should not expect too much. When all men unite on Calvinism or any other religious dogma—when all men are constructed upon the same intellectual scale, with equal powers of analyzing evidence, and divining the truth—then may we have universal concurrence in some one medical doctrine. The great strength of quackery lies in the fact that weak minds require a creed to rest upon, some positive assurance of unfailing certainty. They yield to what is to their mental constitutions a necessity, they must have a distinct and declared theory. Thus it is that they cannot appreciate a purely eclectic organization—they cannot see the beauty of that scheme which leaves the whole field of doctrine and practice open to the judgment of the individual practitioner.

In the mean time there is a steady march toward truth. Knowing its own deficiencies, seeing with clearness the true but laborious path to progress, but recently furnished with the all-important aids of chemistry and an enlightened physiology, the profession never before stood so firm in its own capacities for advancement. It is as impossible to check medical progress as to impede the mechanic arts. It is a science, and while men are found who love science for its own sake, so long will the profession advance.

It can already point to its achievements, to vaccination, to etherization, to that better understanding of the laws of life and of disease which has lengthened human life in all civilized countries. Diseases lose their malignancy. The sore throat of 1734, which caused 61 deaths in a sparsely settled township, finds no record in the mortuary list of 1853. With habits of life less simple and less in accordance with hygienic law, with more numerous cases of more various diseases, with an actual increase of sickness in the town of Hampton, there is a decrease in the number of deaths, and a prolonged average of *human life.*

*Natural Bonesetters.*—There is in the Boston Journal a quaint and somewhat "old grannyish" article on this subject. The style indicates the pen of an old man, while its matter is evidently truthful in intention. A long residence in the same town with Benoni Sweet has furnished to the writer the means of knowing the real merits of this man's claims to skill in the adjustment of bones.

We are surprised to find that this writer, with such opportunities, resorts so largely to hearsay stories to confirm his opinions—which concede to Sweet the power which he claims. Nearly all of the miraculous cases he relates are from second-hand sources and unliable.

He asserts with some show of proof, that the original Sweet who emigrated to Rhode Island, had had the advantages of an English university education, and was probably purely scientific in his mode of operating. To be scientific in the setting of a bone requires many qualifications. The operator should know not only the anatomy of the part, but should understand the lever forces which may be brought to bear upon it. It is probable that all the leverage necessary to be employed, resides within the injured limb, and that a minute knowledge of the various mechanical actions of the muscles, and of the direction in which their force is applied, would obviate the necessity of complicated machinery. But to reduce a fracture well a man must be not only an anatomist and surgeon, but a mechanic.

We have wandered from the matter which we wished to introduce. The writer of the article mentioned, relates the following curious history, without any comment, or apparent consciousness of its importance.

"Many years ago, when I was a student of medicine, I was riding past the house of Capt. Samuel Thompson, of Westerly, R. I., who seeing me, came out and invited me in, saying that the great Dr. Sweet was within and was going to set a bone. I went in accordingly and saw him operate. It was a case of dislocation of the right os femoris from the acetabulum. He operated without any assistant, by placing the patient, a boy of some 10 or 12 years old, upon a truckle bed, lying on his back. *He then elevated the limb, to a right angle, bending it at the joint of the knee, putting his own breast against it, and, making the os femoris act as a lever, gave a sudden push, and did no more. I afterward saw the boy walking the streets.*"

Here we can recognize Dr. Reid's method, which seems to have been wandering unappreciated through New England, no one perceiving its importance until, at last, a surgeon in western New York confers an inestimable benefit upon the profession, by clearly describing, and skillfully performing it. If the profession of New England knew, as they claim to have known, this method, and have kept it all this time to themselves, they deserve the reward of the servant who, having only a single talent, "went and hid it in a napkin."

*Legalized Dissections.*—We are able, at this writing, to inform our readers that the "Bill for the Promotion of Medical Science" has passed the senate of the state, and is now before the assembly. It is now probable that the profession is to obtain its long-sought rights in this matter. The provisions of the bill are sufficiently liberal to secure a sufficient supply of anatomical material for the profession of this state, from within its own boundaries.

This is an important point gained. It is a recognition of the claims of the profession; it acknowledges the injustice of the laws relative to mal-practice, and removes from them the old grievance of requiring anatomical knowledge in the surgeon, while it forbids it to the student.

Perhaps we are holding our celebration before the result of the election is known. It may be that there is not sense or wisdom enough in the lower house, to appreciate the humane and beneficent character of this bill. We do know of some, however, who are pledged to its support, and will give to it their best efforts. Let the profession remember these men for good, and reward them as best it may for their intelligent and honorable course. On the other hand, it is becoming a well-acknowledged principle in political action, that punishment for malfeasance should be as certain as rewards for services.

There is one provision we are most glad to notice. It is that clause which makes it a penal offense to send subjects out of the state to other medical colleges. Each state should supply itself in this matter. The state which cannot furnish sufficient anatomical material for its colleges, should have no schools.

This new law has become a necessity. The difficulty of procuring subjects illegally has been year by year increasing, until the supply has become entirely inadequate. Thus it is said that one of the metropolitan schools suffered, last year, a loss of twenty dollars each, on eighty subjects, making a total of sixteen hundred dollars to be deducted from the income of the school.

In all the schools the embarrassment has been more or less felt. At present, in some of the country schools, it is with great difficulty that any have been obtained. In the government school of Michigan, at Ann Arbor, there has been an almost complete destitution. We are credibly informed that only one dissection class had been organized there at the close of the third month of the term. The lecturer on anatomy had, at that time, no subject, and was at the last accounts waiting the arrival of one procured from a distant city, but which was delayed somewhere upon the route.

Of course this would not have happened in a school relying on its reputation for its support; and we can only look upon it as an indication of what all our schools would be under the same system. But there is an injustice connected with the supply of material from foreign sources. The attempt has been repeatedly made to procure material for distant schools in this city. Now whatever may be the abundance of the supply here, it will be very readily seen that any *emeute* arising here, from the violation of the present law, would fall, not on the heads of those in distant places who profit by it, but would be directed upon the school of this city, and it would be made to suffer for the sins of others, who would laugh at its calamities.

We trust that this will sufficiently account to sundry correspondents of our own for the prompt refusal which was given to their requests, as well as to others who have failed in the attempt to make this city the resurrection ground for western schools. While we of Buffalo have, in every instance, avoided and forbidden the violation of the grave for our own purposes, we shall hardly be willing to knowingly permit others to do that, which would most assuredly react upon the innocent.

Should the bill now before the legislature become a law, we can safely speak for the profession of this state, when we say that it will be rigidly observed by them, and that they would join in condemnation of any violation of it from any source, domestic, or foreign.

*The Cholera.*—From various sources, and from different countries, we hear of the approach of this dreaded epidemic. During the autumnal months it reached England, and immediately manifested itself in the great towns of London, Liverpool and Manchester, as well as in other localities of less importance. At no place was the number of deaths very large, and the disease seems likely to do as it has done before, to lay dormant, in a measure, through the winter, and, it is feared, to break out with its old malignancy at the coming of warm weather.

The public authorities have taken some measures to check the progress of the malady. Among the most important of these is the appointment of medical visitors, whose duty it is to ascertain, and report upon, all nuisances; to visit suspicious localities; to ascertain the health of their inhabitants, and to prescribe for all who may need it. This latter duty is extended to those who may not have applied for relief, although in need of it. Very many of the poor families have, in all these epidemics, manifested a strange apathy in calling for medical aid. This is now avoided by the personal visits of the visitor, who inquires into their health, and prescribes without waiting to be called upon. This measure is apparently doing much to curtail the ravages of the disease, as it insures treatment in that prodromic stage when treatment is most effectual.

In the mean time the cities of Scotland—Edinboro and Glasgow, especially—are suffering to a much greater extent than is England. The same sanatary precautions are not enforced—the "closes" of those towns are, as they ever have been, filthy beyond description—and the epidemic goes on unchecked. It is worthy of notice that the Presbytery of Scotland addressed, last fall, a letter to Viscount Palmerston, imploring Her Majesty the Queen, to appoint a day of fasting and prayer to God, that He might avert the coming pestilence.

The secretary, in reply, pointed out to them the filth of their cities, and urged upon them that they should first cleanse their streets and dwellings, and then, having removed all causes of disease within their reach, should these means be unavailing, they would be able to show sincerity in those prayers, which would be their only remaining resource.

This letter was an eminently sensible, and Christian rebuke to that Mohammedan fatalism, which would fold the hands, and cry "Allah is great!" forgetting that He is a God of Judgment, and not of vengeance; and that it is impious to ask for benefits while we neglect the reformation of glaring sins against life and health.

The cholera is now in Paris. As in England, the number is not large, compared with the immense population of that metropolis, but still enough to demonstrate that the coming of spring will develop it in full force. They do not seem to be more successful in its treatment in Paris than elsewhere. No new facts have been developed, and we are only advanced in its treatment, inasmuch as we know that it is a zymotic disease, and subject to the usual laws of zymosis.

As yet no deaths from cholera have been reported on this side the Atlantic. The emigrant ships coming over, lose hundreds of passengers every trip, from what is supposed to be cholera, but the restoration to purer air upon landing, and the deliverance from the horrors of the steerage, seem to cut short the further development of the disease. That so many deaths occur in these passages is no wonder. The emigrants are huddled into the crowded steerage; in bad weather the hatches are battened down, and the air becomes, from these causes, extremely foul. Add to this, the fact that these people will not come upon deck when they can; that their habits are most shockingly filthy, and that their dead lie side by side with the living until the ship's officers discover them; and it is no mystery that the horrors of an emigrant ship equal those of a slaver on the "middle passage."

Senator Fish, of this state, has introduced a measure into the U. S. Senate, to check, if possible, this sacrifice of life. He has shown in this the qualities of the true statesman, as well as those of the humane gentleman. What he proposes we do not know, but some action of Government is certainly called for in this matter. Quarantines are ineffectual, but if government were to still further limit the legal number of passengers, it would tend to prevent the creation of floating pest-houses to be cast upon our shores.

But so long as our cities are left in a condition of filth which shall generate and nourish disease, it will be impossible to close the inlets, so that it shall not come among us. It is found in England, that the same neighborhoods, and the same houses where cholera was most fatal two years since, are again its chosen habitat. This fact indicates the efficiency of local causes in producing it, and is the strongest argument possible for effort and vigilance on the part of city authorities.

*Effect of Chloroform in inducing narcosis from opium previously taken.* Dr. E. W. Booth, of Miss., reports, in the Southern Medical Journal, a case of delirium tremens, in which he ordered grain doses of morphine to be given every hour. At his second visit his patient was raving violently, and evidently not at all under the influence of the opiates, though he had taken ten grains.

He gave, internally, a half a drachm of chloroform in a little water, which he repeated in ten minutes. Very soon after the second dose, he fell into an apoplectic stupor, "his breathing was not oftener than once in a minute; his eyes turned upward; his pulse about one hundred, full and regular; his respiration was loud and stertorous," and, in short, his patient was instantly and most dangerously narcotized. Several hours of exertion on the part of his physician rescued him from his condition.

In commenting upon this case, the editor of the Southern Journal attributes to chloroform a power of so obtunding the morbid excitement of the nervous system, as to subject it, for the first time, to the full influence of the enormous quantity of morphine which had been administered. He derives a practical conclusion from this case. When large doses of opium have been administered in cases of delirium tremens, or other disease where great tolerance of the drug exists, it is unsafe to follow them with chloroform, as that may at once give efficacy and narcotic power to the whole quantity. He mentions a case of tetanus, where chloroform had been inhaled during the free exhibition of morphine, and says that the patient evidently died narcotized. H.

